# Measuring the Economic Value of the Negative Externality of Livestock Malodor in South Korea

**DOI:** 10.3390/ijerph19159475

**Published:** 2022-08-02

**Authors:** Kwideok Han, Jeffrey Vitale, Yong-Geon Lee, Inbae Ji

**Affiliations:** 1 Department of Institutional Research and Analytics, Oklahoma State University, 203 PIO Building, Stillwater, OK 74078, USA; kwideok.han@okstate.edu; 2 Department of Agricultural Economics, Oklahoma State University, 418 Ag Hall, Stillwater, OK 74078, USA; jeffrey.vitale@okstate.edu; 3 Department of Environment and Resources Research, Korea Rural Economic Institute, 601 Bitgaram-ro, Naju-si 58217, Korea; yglee@krei.re.kr; 4 Department of Food Industrial Management, Dongguk University, 30 Pildong-ro 1-gil, Jung-gu, Seoul 04620, Korea

**Keywords:** livestock malodor, contingent valuation, willingness-to-pay, economic valuation, double-bounded dichotomous choice model

## Abstract

The South Korean livestock industry has increased in scale and production, generating positive impacts on the national economy. However, livestock externalities, primarily malodor, have subsequently led to increased conflicts between producers and affected communities. This study estimated Korean households’ willingness-to-pay (WTP) for government subsidies to help address livestock malodor using a contingent valuation method (CVM) derived from a double-bounded dichotomous choice model. The annual average household WTP was estimated at 29,206 Korean won (KRW) (USD 25). This was slightly higher than the respondents’ self-reported average amount of KRW 25,457 (USD 22). The estimated economic value nationally is KRW 628 billion (USD 546 million) annually, for a total of KRW 3.14 trillion (USD 2.73 billion) over a proposed five-year period. The public’s estimated WTP can be leveraged to improve livestock management practices, more efficient waste disposal techniques, and improved husbandry methods to address conflicts between producers and surrounding communities.

## 1. Introduction

Korea’s livestock industry has grown through the scaling-up and intensification of livestock operations achieved by increased efficiency and policy support [1]. The livestock industry plays a positive role in Korea’s national economy by increasing the income of livestock farmers, enhancing dietary nutrition, job creation, and reducing Korea’s dependence on meat imports. Despite this positive role, environmental problems, such as water pollution, greenhouse gas emissions, excess soil nutrients, and malodor from livestock manure, are constraining the growth of the livestock industry and causing social conflicts with local communities. Such concerns are commonplace in agriculture throughout the world but have grown in magnitude and severity in recent decades with the continued development of large-scale confined feeding operations [2,3,4,5,6].

One of the negative aspects of the livestock industry causing conflict and drawing a large number of complaints from local residents is livestock malodor associated with manure [7,8,9,10,11,12]. In Korea, livestock odor complaints have increased significantly from 2838 cases in 2014 to 12,631 cases in 2019, an increase of 19.2% to 30.9% of the total odor complaints (Ministry of Environment (MOE); http://stat.me.go.kr/portal/main/indexPage.do, last accessed on 24 January 2021). Environmental problems in Korea caused by the livestock industry have led to stricter regulations, as legislated by the “Act on the Management and Use of Livestock Manure” (MOE; https://me.go.kr/home/web/index.do?menuId=64, last accessed on 10 May 2022). However, in 2017, 329 out of 3075 livestock excreta discharge facilities violated the government’s protocols, and 18 of these facilities exceeded the critical point for effluent. Additionally, zoning requirements announced by the Korean Ministry of Environment have been loosely adhered to by local administrations, often resulting in residential areas in very close proximity to livestock facilities.

For the sustainable development of the Korean livestock industry, it is essential to strengthen the economic competitiveness and simultaneously reduce its environmental footprint. To achieve this, the Korean government has promoted and subsidized more environmentally friendly measures to mitigate the impacts of livestock excreta [1]. Still, the demand to improve livestock environmental policies has increased, and environmental regulations surrounding the livestock industry continue to be strengthened. For example, compost maturity standards were strengthened in 2020, mandating facilities over 1500 m^2^ to distribute compost only in later stages, and in 2021, regional nutrient management systems were adopted in agricultural areas.

Malodor problems caused by the livestock industry can be considered as a non-marketable environmental product and a part of a “provisional” ecosystem service [13,14]. Ecosystem services are not always beneficial and, in the case of livestock malodor, are external costs lacking valuation from market-based outcomes [13,15,16]. Though difficult and controversial to measure, reports of economic damage to communities by malodor and related nuisances have been presented in the literature [17,18]. Public perceptions of the negative externalities in the livestock industry have been elicited to place an economic value on nuisances such as malodor associated with confined livestock operations [19].

Previous studies have estimated how individuals value various aspects of agriculture, including their willingness-to-pay (WTP) for research and development (R&D), investments in rural areas, and mitigating both positive and negative externalities in agriculture [14,20]. The contingent valuation method (CVM) is considered the most appropriate and most commonly utilized method to estimate how individuals value resolving externalities [21,22]. In Korea, Ji et al. [23] was one of the first to apply this method to estimate the economic value of externalities of the Korean livestock industry. The WTP for agricultural R&D and investments in rural areas ranged between 8–26 trillion Korean won (KRW) (nominal currency) when scaled from the household to the national level [23] and references therein.

In this study, we measure the economic value of the negative externality of livestock malodor in South Korea. Results provide estimates of the perceived economic importance as determined from respondents’ WTP. This study uses the CVM with a double-bounded dichotomous-choice model. The estimated WTP can be useful to support government policies and subsidies to further alleviate environmental impacts of the livestock industry. This study proceeds with a description of the survey design, followed by the estimation methodology of the single- and double-bounded models used in CVM. Next is a summary of the survey data followed by a discussion of the results. The paper ends with conclusions based on policy implications for stakeholders in the South Korean livestock industry. 

## 2. Materials and Methods

### 2.1. Survey Design

An online questionnaire was developed to ascertain the awareness of and the willingness-to-pay for addressing malodor generated by the livestock industry. A total of 1000 households were surveyed online from 21–30 July 2021. The survey sample was randomly drawn from a survey panel, managed by an independent survey company, of more than 500,000 people. The sample of 1000 households is representative of the population of Korea, based on the 2019 Population and Housing Census, in terms of gender, age, monthly income, proportion living in urban/rural areas, and geographical region.

**Figure 1:**
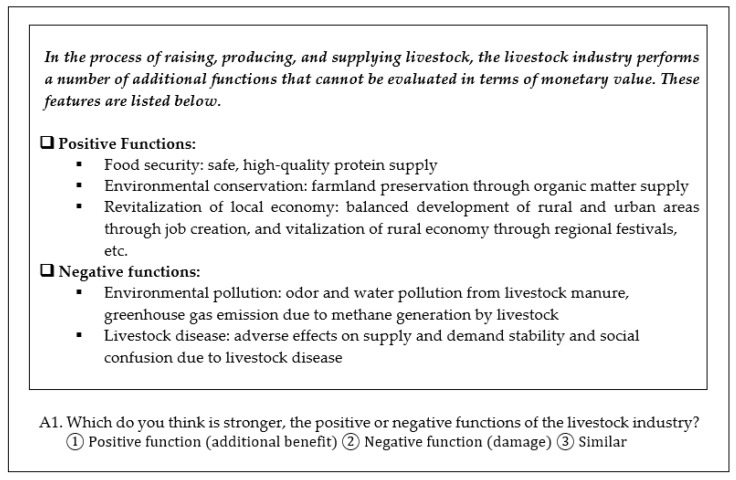
Sample question for a respondent’s opinion on the aspects of the livestock industry.

The online questionnaire first elicited the demographic characteristics of the respondents including education, marital status, household size, and average monthly income. Next, information about the positive and negative aspects of the livestock industry upon the economy and environment was presented to the respondents prior to asking their awareness of these issues. Positive aspects emphasized food security, environmental conservation, and local/regional economic revitalization. Negative aspects emphasized environmental pollution, both air and water, as well as the societal and economic impacts of livestock disease outbreaks. Respondents were then asked which they considered had a stronger effect, the positive or negative aspects of the livestock industry, or whether these were similar in effect (Figure 1).

Then, the respondents were presented with additional information about the negative environmental issues caused by the livestock industry. This included descriptions of malodor and increased greenhouse gas emissions because of livestock methane production, as well as water pollution due to an excess supply of soil nutrients (nitrogen and phosphorus) from manure and chemical fertilizers and the direct contamination of water resources from livestock excreta discharge facilities. The questionnaire then focused upon the respondent’s level and degree of experience with malodor associated with the livestock industry. Respondents were asked about their personal experience with malodor, including the frequency of occurrence, when during the year malodor issues most often occurred, where it occurred, what type of livestock it was associated with, which type of livestock or agricultural facility was involved, and whether or not the livestock facility should be eliminated from the area.

The negative and positive effects of the livestock industry were again summarized, and the need for government subsidies to address malodor problems was presented. Respondents were provided with an explanation of why government subsidies were necessary to manage the malodor issues of the livestock industry. This provides information upon which respondents are able to determine whether the government can assist the livestock industry in addressing these issues. The explanation was followed by a direct question asking respondents about their household’s WTP a certain stated amount so government subsidies could be used to help alleviate the malodor issues of the livestock industry. 

**Figure 2:**
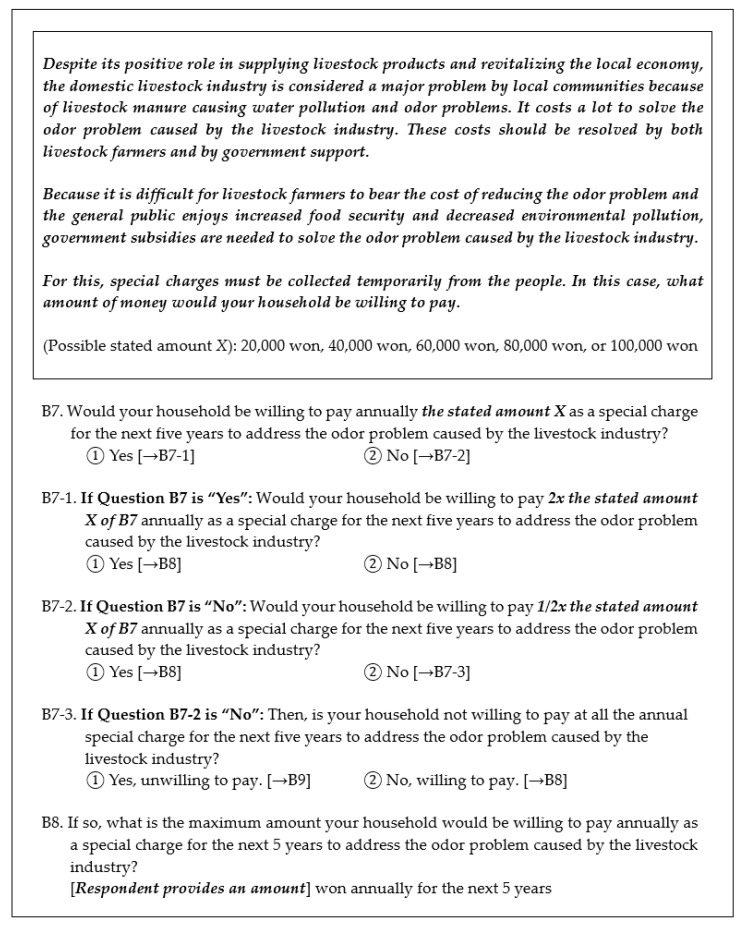
Sample double-bounded dichotomous choice questions for a respondent’s WTP.

To address malodor issues in the livestock industry, the WTP based upon a stated amount was determined by a CVM questionnaire. The CVM survey was reviewed by a panel of fifteen (15) experts in the livestock field who assisted in refining and developing the questions and WTP amounts used. The 1000 respondents were randomly divided into five equal groups of 200 respondents each. For each group, the initial WTP question presented one of five stated amounts ranging from KRW 20,000 in group 1 to KRW 100,000 in group 5 in increasing increments of KRW 20,000 (Figure 2). 

A double-bounded dichotomous choice questioning method was used to determine the WTP to address the malodor issue. If the respondent’s answer was “yes” to the first question, accepting the initial stated amount, then the stated amount in the follow-up question was double (2X) the initial stated amount. If the respondent’s answer was “no” to the first question, rejecting the initial stated amount, the stated amount in the follow-up question was halved (½X) the initial amount.

To further determine the bounds of WTP, respondents that answered both of the dichotomous questions were asked an additional question regarding the maximum amount they would be willing to pay annually for five years to address the malodor issue. The respondent was provided an opportunity to type in an amount. For respondents that answered “yes–yes”, if the respondent provided a maximum amount, this needed to be greater than or equal to the 2X stated amount. For “yes–no” answers, if the respondent provided an answer to this additional question, the amount needed to be between the initial amount X and the 2X stated amount. For respondents answering “no–yes”, answers to the additional question needed to be between ½X and X.

For respondents that answered “no–no” to both the initial and follow-up questions, an additional question was posed as to whether they were sure that their household was unwilling to pay any amount. A “Yes” answer was that they were unwilling to pay any amount, and this was indicated by an amount of KRW zero. A “No” answer to this question indicated a WTP, and the respondent was then provided an opportunity to type in an amount in KRW.

### 2.2. Econometric Modeling

To analyze the CVM survey responses, we estimate both the single- and double-bounded dichotomous choice models. In the single-bounded model, the respondents’ answers, “yes” or “no”, were analyzed as to whether or not they would accept the stated amount to address livestock malodor. For the double-bounded choice models, respondents’ answers for the first question and answers to a second question, contingent on the answer to the first question, were analyzed.

The *WTP* for addressing livestock malodor for a respondent *i* can be directly estimated as:

(1) $$WTP_{i}=x_{i}\beta +\varepsilon_{i},$$

where $WTP_{i}$ is the ith respondent’s true WTP, $x_{i}$ is a vector of individual characteristics, β is a vector of parameters to be estimated, and $\varepsilon_{i}$ is an independently and identically distributed normal error term with mean zero and variance $\sigma^{2}$.

#### 2.2.1. Single-Bounded Model

The single-bounded model estimates respondents’ WTP based on a single dichotomous choice CVM question [24]. If an individual *i* responds “yes” to the stated amount, $t_{i}$, the range of WTP is lower bounded by $t_{i}$ ($t_{i}\leq WTP_{i}<\infty$). A “no” response is upper bounded by $t_{i}$ ($0\leq WTP_{i}<t_{i}$). Because there is only a single response, a large sample is required for an efficient estimate of WTP [25].

The probability of a “yes” or “no” response can be represented by:

(2) $$\pi^{y}\left(t_{i}\right)=Pr\left(t_{i}\leq WTP_{i}\right)=1-\Phi \left(\frac{t_{i}-x_{i}\beta }{\sigma }\right)$$

(3) $$\pi^{n}\left(t_{i}\right)=Pr\left(t_{i}>WTP_{i}\right)=\Phi \left(\frac{t_{i}-x_{i}\beta }{\sigma }\right)$$

where π represents the response probabilities and Φ(·) is the standard normal cumulative density function of the respondent’s *WTP*.

Then, the log-likelihood of the single-bounded model for *N* respondents is specified as:

(4) $$\ln L=\overset{N}{\underset{i=1}{\sum }}\left\{d_{i}^{y}\ln\left[1-\Phi \left(\frac{t_{i}-x_{i}\beta }{\sigma }\right)\right]+d_{i}^{n}\ln\Phi \left(\frac{t_{i}-x_{i}\beta }{\sigma }\right)\right\},$$

where $d_{i}^{y}$ is 1 if the individual i responses “yes” and 0 if “no”, while $d_{i}^{n}$ is 1 if the individual i responses “no” and 0 if “yes”.

#### 2.2.2. Double-Bounded Model

The double-bounded model estimates respondents’ WTP based on the initial and follow-up dichotomous choice CVM questions [26,27]. The follow-up question is contingent upon the response to the first question. If an individual responds “yes” to the initial stated amount, $t_{i}$, then the follow-up stated amount, $t_{i}^{2}$, is higher $\left(t_{i}<t_{i}^{2}\right)$. If an individual responds “no” to the initial stated amount, $t_{i}$, then the follow-up stated amount, $t_{i}^{1}$, is lower $\left(t_{i}^{1}<t_{i}\right)$. The follow-up question results in a more refined range of the respondents’ unobserved true WTP.

Thus, there are four possible outcomes: (a) “yes–yes”; (b) “no–no”; (c) “yes–no”; and (d) “no–yes”. The range of WTP is determined by each outcome. If respondent *i*’s answer is “yes–yes”, the range of WTP is lower bounded by $t_{i}^{2}$ ($t_{i}^{2}\leq WTP_{i}<\infty$). If respondent *i*’s answer is “no–no”, the range of WTP is upper bounded by $t_{i}^{1}$ ($0\leq WTP_{i}<t_{i}^{1}$). When respondent *i*’s answer is “yes–no”, the range of WTP is lower bounded by $t_{i}$ and upper bounded by $t_{i}^{2}$ ($t_{i}\leq WTP_{i}<t_{i}^{2}$), while when respondent *i*’s answer is “no–yes”, the range of WTP is lower bounded by $t_{i}^{1}$ and upper bounded by $t_{i}$ ($t_{i}>WTP_{i}\geq t_{i}^{1}$).

Following Hanemann et al. [25], the probabilities of these outcomes, $\pi^{yy}$, $\pi^{nn}$, $\pi^{yn}$, and $\pi^{ny}$ are as follows:

(5) $$\pi^{yy}\left(t_{i},\;t_{i}^{2}\;\right)=Pr\left(t_{i}\leq WTP_{i},\;t_{i}^{2}\leq WTP_{i}\right)=Pr\left(t_{i}^{2}\leq WTP_{i}\right)=1-\Phi \left(\frac{t_{i}^{2}-x_{i}\beta }{\sigma }\right),$$

(6) $$\pi^{nn}\left(t_{i},\;t_{i}^{1}\;\right)=Pr\left(t_{i}>WTP_{i},\;t_{i}^{1}>WTP_{i}\right)=Pr\left(t_{i}^{1}>WTP_{i}\right)=\Phi \left(\frac{t_{i}^{1}-x_{i}\beta }{\sigma }\right),$$

(7) $$\pi^{yn}\left(t_{i},\;t_{i}^{2}\;\right)=Pr\left(t_{i}\leq WTP_{i}\leq t_{i}^{2}\right)=\Phi \left(\frac{t_{i}^{2}-x_{i}\beta }{\sigma }\right)-\Phi \left(\frac{t_{i}-x_{i}\beta }{\sigma }\right),$$

(8) $$\pi^{ny}\left(t_{i},\;t_{i}^{1}\;\right)=Pr\left(t_{i}\geq WTP_{i}\geq t_{i}^{1}\right)=\Phi \left(\frac{t_{i}-x_{i}\beta }{\sigma }\right)-\Phi \left(\frac{t_{i}^{1}-x_{i}\beta }{\sigma }\right),$$

where π represents the response probabilities and Φ(·) is the standard normal cumulative density function of the respondent’s *WTP*.

Equations (5) and (6) improve the single bound by refining the lower and upper bounds of WTP values, whereas Equations (7) and (8) elicit WTP values within finite bounded intervals. Therefore, the WTP estimates of the double-bounded model are statistically more efficient than those of the single-bounded model because of the improved reliability of the WTP estimates [28].

The log-likelihood of the double-bounded model for *N* respondents is specified as:

(9) $$\begin{array}{l} \ln L_{i}=\overset{N}{\underset{i=1}{\sum }}\{d_{i}^{yy}\ln\left[1-\Phi \left(\frac{t_{i}^{2}-x_{i}\beta }{\sigma }\right)\right]+d_{i}^{nn}\ln\Phi \left(\frac{t_{i}^{1}-x_{i}\beta }{\sigma }\right)\\ \;+d_{i}^{yn}\ln\left[\Phi \left(\frac{t_{i}^{2}-x_{i}\beta }{\sigma }\right)-\Phi \left(\frac{t_{i}-x_{i}\beta }{\sigma }\right)\right]\\ \;+d_{i}^{ny}\ln\left[\Phi \left(\frac{t_{i}-x_{i}\beta }{\sigma }\right)-\Phi \left(\frac{t_{i}^{1}-x_{i}\beta }{\sigma }\right)\right]\}, \end{array}$$

where $d_{i}^{yy}$, $d_{i}^{nn}$, $d_{i}^{yn}$, and $d_{i}^{ny}$ are binary indicator variables with values of 1 or 0 for the corresponding respondent probabilities. 

The maximum likelihood (ML) estimator can be used to estimate *β* and *σ* for both the single- and double-bounded models. The mean *WTP* is calculated as:

(10) $$E\left(WTP\right)=\bar{x}\hat{\beta },$$

where $\bar{x}$ is a vector of the sample averages of the explanatory variables.

We estimated respondents’ WTP with only a constant as an explanatory variable, and also with additional explanatory variables, including demographic characteristics, that may affect valuation. The confidence intervals for the estimates of WTPs are computed using the simulation method of Krinsky and Robb [29]. The *R* package ‘DCchoice’ was utilized to analyze the CVM survey responses [30].

## 3. Results and Discussion

Summary statistics and variable definitions are reported in Table 1. The average age of the respondents was 44 years old, ranging from 20 to 69 years old, and the average monthly household income was KRW 4,326,000. Just over half of the respondents (51%) were male, and a majority (65%) of respondents were married with the average household containing three people. Two-thirds (66%) of the respondents believe that the livestock industry plays a positive role compared to slightly less than 8% who answered that the negatives outweigh the positives, while the remaining 26% answered that the positive and negative aspects were similar. Among the 1000 respondents, 80% resided in urban areas, less than half (48%) had agricultural experience, and 87% answered that livestock malodors were an issue.

**Figure 3:**
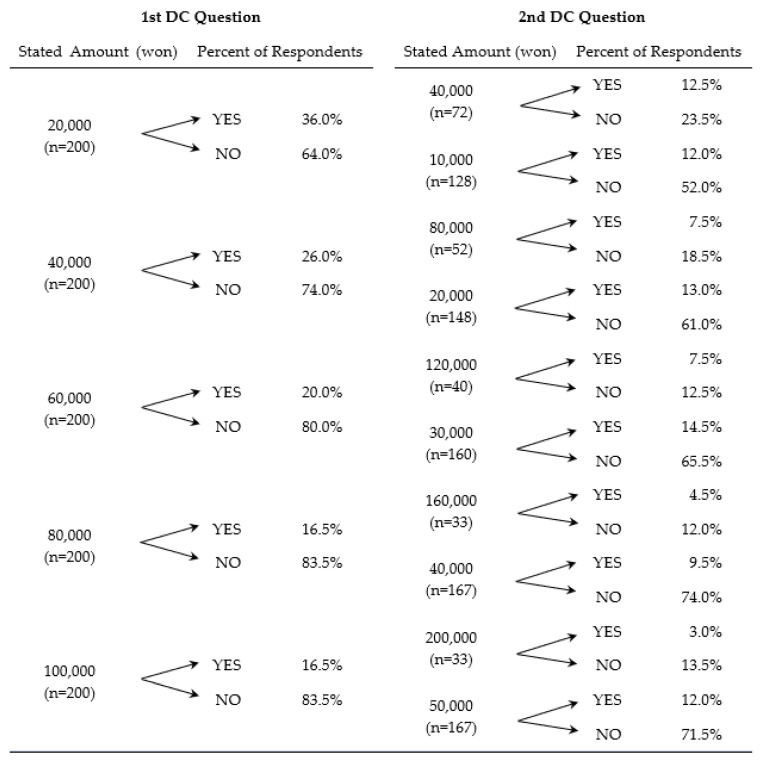
Summary responses to double-bounded dichotomous choice questions. All percentages are calculated from the total number of respondents for each stated amount (n = 200).

The summary statistics of responses to the double-bounded dichotomous choice questions are presented in Figure 3. Of the 1000 respondents, 453 indicated a WTP in at least one of the questions: the initial, follow-up, and/or additional question posed that allows the respondent to provide their own amount. For the initial and follow-up questions: a total of 70 respondents answered “yes–yes”, 160 answered “yes–no”, 122 answered “no–yes”, and 648 answered “no–no”. Of the 648 respondents that answered “no–no”, 101 respondents, when given the additional question B7-3 (Figure 2), answered “No” that they were willing to pay and then provided an amount in KRW. The other 547 “no–no” respondents answered, “Yes” that their household has no intention to pay, and these were assigned a value of KRW zero. An additional question asked these 547 respondents for the best reason explaining why the respondent was not willing to pay. The three top responses, out of the nine choices provided, were that it was a problem that the livestock industry should resolve on its own (35.1%), that it should be covered by taxes already paid (31.3%), and that the household could not afford to pay anything additional (15.5%).

Some interesting trends were noted. As the initial stated WTP amounts increased, the acceptance rate (a “yes” response) decreased from 36% for the lowest initial stated amount of KRW 20,000 to 16.5% for each of the two higher stated amounts, KRW 80,000 and KRW 100,000. Additionally, with the exception of the first group (where the initial stated amount was KRW 20,000), the percentage of respondents answering “no–yes” was approximately double that of the respondents answering “yes–yes”. Among the 101 respondents that initially answered “no–no” but then indicated a WTP to a third question, as the initial stated amount increases, the average amount indicated by the respondents increases. “No–no” respondents with an initial stated amount of KRW 20,000 (n = 13) averaged about KRW 5000, whereas “no–no” respondents that had initial stated amounts of KRW 80,000 and KRW 100,000 (n = 26 and 21, respectively) averaged about KRW 14,000. It appears that the initial amount stated could potentially be biasing the amount that respondents would ultimately be willing to pay even when they initially indicated “no–no” to the first two dichotomous choice questions.

### 3.1. Willingness-to-Pay Estimates

The WTP estimates for addressing malodor issue of the livestock industry using the single- and double-bounded dichotomous choice models without and with explanatory variables are reported in Table 2 and Table 3, respectively. In the models without explanatory variables, the estimated coefficients on the stated WTP amount are both significant and negative, indicating that as the stated amount increases the respondents’ WTP decreases by about 8% for the single-bounded model and about 13% for the double-bounded model (Table 2). The respondents’ average WTP from the single-bounded model was KRW 34,341 with a 95% confidence interval (CI) of KRW 29,568 and KRW 43,730. The respondents’ average WTP from the double-bounded model was KRW 29,873 with a 95% CI of KRW 26,917 and KRW 33,093. The estimated WTP of the double-bounded model is more refined than that of the single-bounded model as expected.

When explanatory variables were included, the estimated coefficients on the stated WTP amount in the single- and double-bounded models (Table 3) were consistent with what was found previously without the explanatory variables (Table 2). The respondents’ average WTP from the single-bounded model was KRW 33,695 with a 95% CI of KRW 28,648 and KRW 43,808. The respondents’ average WTP from the double-bounded model was KRW 29,206 with a 95% CI of KRW 26,149 and KRW 32,309. Although there may be concerns of the inconsistency of the estimates of WTP between the initial and follow-up questions [31] (however, see [32]), our results are consistent. The average WTP in both the single-bounded models, without and with explanatory values, were essentially identical at about KRW 34,000. In the double-bounded models, without and with the explanatory variables, the average WTPs were slightly different at KRW 30,000 and KRW 29,000, respectively. The double-bounded model is preferred as it provides a statistically efficient estimate of the WTP [33].

All parameter estimates of the double-bounded model maintained their same effect and significance was nearly identical, as compared to the single-bounded model (Table 3). The explanatory variables significantly related to WTP in both the single- and double-bounded models were household monthly income, household size, current residence (urban/rural), and any agricultural experience. Respondents with higher monthly income, smaller household size, currently living in rural areas, and with agricultural experience were willing to pay more. This is consistent with expectations, as smaller sized households with higher incomes may have more disposable income to pay, and those with agricultural experience or living in rural areas may be familiar with malodors associated with the livestock industry.

The parameter estimates of three variables differed in significance between the single- and double-bounded models. In the single-bounded model, hometown and livestock industry perception have a significant positive effect on WTP at the 10% level. Whereas in the double-bounded model, it is noteworthy that gender has a significant negative effect on WTP at the 1% level, indicating that males are less willing to pay than females. Variables that were not significant in either model included malodor experience. This lack of significance for malodor experience is likely due to the large number of respondents (87.1%) who had experienced the malodor making it difficult to detect any effect.

The findings from this study shared few similarities with a previous study [23] estimating the WTP to support the Korean livestock industry, although this previous study did not specifically address malodor issues. In the previous study [23] and this study, income had a significant positive effect on the WTP. The perception of livestock industry in Ji et al. [23] was found as having a significant positive effect on the WTP, but in this study, it was only significant in the single-bounded model at the 10% level. Other factors found significant in this study, as compared with Ji et al. [23], were the effects of gender (in the double-bounded model only), hometown (in the single-bounded model only), and the current residence (urban/rural) and agricultural experience (Table 3).

Results from this study are consistent with findings from previous research estimating the WTP for agricultural and environmental services including resolving externalities such as malodor. The strongest agreement among studies is the significant negative effect of initial stated amount [34,35]. The negative relationship is not surprising since, as the WTP amount increases, it is less likely to be accepted. Similarly, income was found to have a significant positive effect on WTP as found in previous studies [35]. Household size has a significant negative effect, and this may be attributable to the availability of disposable income [36]. Our study found a negative effect of the location of the current residence (urban/rural) indicating that urban respondents have a significantly lower WTP for agricultural services, also consistent with what has been reported in other studies [37]. When considering gender, females were found to be significantly willing to pay more than males as previously described [38]; however, other studies report that gender has no significant effect [21,35,39]. 

The most noteworthy inconsistency with prior WTP studies, including Ji et al. [23], was the lack of explanatory power of the livestock industry perception variable (Table 3). The vast majority of previous WTP studies have found attitudes and perceptions held by respondents to have significant effects on WTP for agricultural amenities [21,40,41,42]. In our study, it would have been expected that respondents with a favorable attitude of the Korean livestock industry would have a significant positive effect on WTP, as had been found in Ji et al. [23]. The lack of significance could be explained by malodor creating a negative perception of the livestock industry and a greater incentive for households to make investments to address the malodor externality. 

### 3.2. Economic Value to Alleviate Livestock Malodor

The economic value to alleviate the malodor concerns of the livestock industry nationwide was calculated by multiplying the estimated average WTP by the total number of households in Korea. The number of households in Korea were 21,484,785 in 2020 (Statistics Korea; https://kostat.go.kr/portal/korea/kor_nw/1/2/2/index.board, accessed on 20 January 2022). The economic values were calculated from the average WTP estimates of the single- and double-bounded models without and with explanatory variables as KRW 738 billion, KRW 724 billion, KRW 642 billion, and KRW 628 billion annually for each model, respectively (Table 4). Over 5 years, the time that the government subsidies would exist, the amounts range between KRW 3.14 to KRW 3.69 trillion. The economic values estimated are higher than those estimated in the previous study [23]; however, direct comparisons are challenging as the two studies investigated different aspects of support for the livestock industry. 

## 4. Conclusions

The Korean livestock industry has increased production though increased cost efficiency and productivity over the last few decades. Successful gains in production have been accompanied by increasing conflict with surrounding communities due to livestock malodor and similar externalities. Our study implies that the Korean public would support investment in policies and technologies to alleviate livestock externalities. The average annual household WTP was estimated at KRW 29,206. This amount is close to the self-reported annual average of KRW 25,457. The nationwide total economic value estimated to address livestock malodor over five years is KRW 3.14 trillion–3.69 trillion. 

Our findings suggest that Korean stakeholders and environmental advocacy groups should consider improving public awareness of livestock externalities. A majority of the survey respondents (66%) have a positive perception of the livestock industry, yet this favorability was not captured by a household’s WTP to address malodor issues (i.e., respondents’ opinion on the livestock industry had no significant effect on WTP). Although a majority of survey respondents (87%) had personally experienced livestock malodor, less than half (45%) expressed a positive WTP for alleviating livestock malodor issues. Alternatively, the majority of respondents not willing to pay (82%) believe that the livestock malodor problem should be solved internally by the livestock industry, financed using already available tax revenue, or simply indicated they could not afford an additional tax. Further educating the public about the costs and benefits of alleviating livestock externalities may foster greater support for WTP.

If the Korean government acts to actively solve the environmental problems caused by the livestock industry, particularly livestock malodor, policy makers will need to formulate strategies that meaningfully and cost-effectively mitigate the conflict between the livestock industry and affected communities. The successful resolution of livestock malodor will require increased investments in new technology and management practices. Future research will need to assess the feasibility of these investments by estimating the costs of introducing new animal husbandry methods, waste disposal techniques, and technologies on livestock farms. The results of this study determining the public’s WTP for government subsidies to mitigate the negative externalities of the livestock industry is useful to guide policy makers’ decision-making.

## Figures and Tables

**Table 1:** Variable Definition and Summary Statistics (*N* = 1000).

Variable	Definition	Mean	Std. Dev.
Gender	1 if male; 0 if male	0.510	0.500
Age	Age in years	44.457	13.408
Income	Household monthly income level in KRW thousands 1 = less than 2000; 2 = 2000 to 2999; 8 = 8000 to 8999; 9 = more than 9000	4.326	2.339
Marriage	1 if married; 0 if single	0.654	0.476
Household size	Number of people in the household	3.008	1.165
Education	Education level 1 = Middle school graduate; 2 = High school graduate; 3 = Bachelor’s degree; 4 = Graduate degree	2.843	0.591
Current residence	1 if urban; 0 rural	0.800	0.400
Hometown	1 if urban; 0 rural	0.689	0.463
Agricultural experience	1 if yes; 0 otherwise	0.481	0.500
Livestock industry perception	1 if positive; 0 otherwise	0.661	0.474
Malodor experience	1 if yes; 0 otherwise	0.871	0.335

**Table 2:** Estimates of WTP for addressing malodor issue without explanatory variables.

Variables	Single-Bounded Model		Double-Bounded Model	
	Coefficient	*t*-Value	Coefficient	*t*-Value
Constant	−0.274 ***	−2.747	−0.044	−0.902
WTP amount (KRW)	−0.081 ***	−5.078	−0.126 ***	−19.721
lnL	−525.981		−1054.161	
Mean WTP (KRW/household)	34,341		29,873	
(95% Confidence interval)	(29,568, 43,730)		(26,917, 33,093)	

Notes: (1) At the time of the survey, USD 1 is approximately equal to KRW 1150. (2) Triple asterisks (***) represents significance at the 1% level.

**Table 3:** Estimates of WTP for addressing malodor issue with explanatory variables.

Variables	Single-Bounded Model		Double-Bounded Model	
	Coefficient	*t*-Value	Coefficient	*t*-Value
Constant	−0.199	−0.578	0.189	0.651
WTP amount (KRW)	−0.079 ***	−4.938	−0.128 ***	−19.744
Gender (Male = 1)	−0.080	−0.874	−0.220 ***	−2.760
Age	−0.004	−0.943	−0.006	−1.578
Income	0.051 **	2.436	0.039 **	2.111
Marriage (Married = 1)	0.153	1.112	0.172	1.448
Household size	−0.100 **	−2.242	−0.075 **	−1.952
Education	−0.028	−0.358	−0.022	−0.321
Current Residence (Urban = 1)	−0.274 ***	−2.676	−0.149 *	−1.702
Hometown (Urban = 1)	0.184 *	1.773	0.042	0.475
Agricultural experience (Yes = 1)	0.169 *	1.802	0.199 **	2.450
Livestock industry perception (Positive = 1)	0.165 *	1.669	0.139	1.641
Malodor experience (Yes = 1)	0.062	0.434	0.033	0.270
lnL	−514.882		−1041.065	
Mean WTP (KRW/household)	33,695		29,206	
(95% Confidence interval)	(28,648, 43,808)		(26,149, 32,309)	

Notes: (1) At the time of the survey, USD 1 is approximately equal to KRW 1150. (2) Single, double, and triple asterisks (*, **, ***) represent significance at the 10%, 5%, and 1% levels, respectively.

**Table 4:** Estimated economic value of livestock malodor (2020-based).

Variables	Without Explanatory		With Explanatory	
	Single	Double	Single	Double
Mean *WTP* (KRW/household)	34,341	33,695	29,873	29,206
1 Year (KRW billion)	738	724	642	628
5 Year (KRW billion)	3689	3620	3209	3137

Notes: (1) The number of Korean households was 21,484,785 in 2020. (2) At the time of the survey, USD 1 is approximately equal to KRW 1150.

## Data Availability

Data are available upon reasonable request from the corresponding author.
